# Reliability of ultrasound hepatorenal index and magnetic resonance imaging proton density fat fraction techniques in the diagnosis of hepatic steatosis, with magnetic resonance spectroscopy as the reference standard

**DOI:** 10.1371/journal.pone.0255768

**Published:** 2021-08-12

**Authors:** Bien Van Tran, Kouichi Ujita, Ayako Taketomi-Takahashi, Hiromi Hirasawa, Takayuki Suto, Yoshito Tsushima

**Affiliations:** 1 Department of Diagnostic Radiology and Nuclear Medicine, Gunma University, Graduate School of Medicine, Maebashi, Gunma, Japan; 2 Department of Radiology, Gunma University Hospital, Maebashi, Gunma, Japan; McLean Hospital, UNITED STATES

## Abstract

**Purpose:**

To evaluate the reliability of ultrasound hepatorenal index (US-HRI) and magnetic resonance imaging proton density fat fraction (MRI-PDFF) techniques in the diagnosis of hepatic steatosis, with magnetic resonance spectroscopy proton density fat fraction (MRS-PDFF) as the reference standard.

**Materials and methods:**

Fifty-two adult volunteers (30 men, 22 women; age, 31.5 ± 6.5 years) who had no history of kidney disease or viral/alcoholic hepatitis were recruited to undergo abdominal US, MRI, and MRS examinations. US-HRI was calculated from the average of three pairs of regions of interest (ROIs) measurements placed in the liver parenchyma and right renal cortex. On MRI, the six-point Dixon technique was employed for calculating proton density fat fraction (MRI-PDFF). An MRS sequence with a typical voxel size of 27 ml was chosen to estimate MRS-PDFF as the gold standard. The data were evaluated using Pearson’s correlation coefficient and receiver operating characteristic (ROC) curves.

**Results:**

The Pearson correlation coefficients of US-HRI and MRI-PDFF with MRS-PDFF were 0.38 (*p* = 0.005) and 0.95 (*p*<0.001), respectively. If MRS-PDFF ≥5.56% was defined as the gold standard of fatty liver disease, the areas under the curve (AUCs), cut-off values, sensitivities and specificities of US-HRI and MRI-PDFF were 0.74, 1.54, 50%, 91.7% and 0.99, 2.75%, 100%, 88.9%, respectively. The intraclass correlation coefficients (ICCs) of US-HRI and MRI-PDFF were 0.70 and 0.85.

**Conclusion:**

MRI-PDFF was more reliable than US-HRI in diagnosing hepatic steatosis.

## Introduction

Nonalcoholic fatty liver disease (NAFLD) is the most common liver disorder. A meta-analysis reported a prevalence of 24% in the worldwide population [1]. NAFLD is also considered an important cause of fibrosis progression, nonalcoholic steatohepatitis (NASH), and hepatocellular carcinoma (HCC) [2]. Based on the literature, NAFLD has shown a strong association with coronary artery disease, osteoporosis, metabolic syndrome [3], and rheumatoid arthritis [4]. The prevalence of NAFLD varies with age, gender, and weight status [5]. Early detection and quantification of hepatic steatosis play an important role in treatment because NAFLD can be treated by control of diabetes, weight loss or lifestyle modification [6].

Liver biopsy is still described as the reference standard for quantifying liver fat content [7]. However, liver biopsy is invasive, with risk of bleeding and other miscellaneous complications, and also has potential for sample bias and inter- and intra-observer variability [7]. A noninvasive and robust method of hepatic steatosis measurement is necessary not only for early detection of hepatic steatosis, but also for monitoring during treatment. Ultrasound (US) is a widely used noninvasive method of assessing fatty liver disease, particularly as a screening tool, because of its low cost, safety and accessibility. The ratio between echogenicity of the liver tissue and renal cortex, called the hepatorenal index (US-HRI), has been commonly used to estimate the degree of steatosis. This ratio is positively correlated with the fat percentage [8]. However, US-HRI has limitations such as variation of HRI values among machines and operators.

MR scanners provide additional noninvasive alternatives for hepatic steatosis measurements by directly quantifying fat content fraction based on the difference in resonance frequencies between water protons and fat protons. Non-invasive magnetic resonance spectroscopy (MRS) providing proton density fat fraction (MRS-PDFF) has been considered an alternative method for evaluating liver fat content. This method seems to be reasonable and is a potential alternative for quantifying liver fat, given that it has actually been shown to be very accurate in comparison to histological diagnosis [9]. However, performing this technique requires the addition of a special software package usually not available by default. It is also a time-consuming technique, which also hinders its widespread use.

Magnetic resonance imaging proton density fat fraction (MRI-PDFF) is a newer technique proposed in the diagnosis of hepatic steatosis. This technique can be considered a hybrid methodology, as it combines the advantages of complex-based fitting and magnitude-based fitting techniques to estimate fat fraction. The multi-echo adaptive fitting technique uses the Levenberg-Marquardt fitting algorithm to solve for the values of water and fat signal intensity. A multi-step nonlinear fitting procedure is then performed to adaptively update the fat and water signal fractions based on magnitude signal equations with a multi-peak fat spectral model [10]. The major advantages of this technique over MRS are that (1) it is technically easier to implement, (2) the software package needed is commonly available on conventional MRI units, and (3) the examination time is short (less than 15 minutes). We suspect this technique to be a potential replacement for US, or even a first line tool for diagnosis and management of hepatic steatosis.

The purpose of this study was to evaluate the reliabilitiy of fat quantification by US (US-HRI) and MRI-PDFF techniques in the diagnosis of hepatic steatosis, with MRS-PDFF as the reference standard.

## Materials and methods

### Subjects

Adult volunteers having no known hepatic nor renal disease were randomly recruited over a period of 19 months (Nov. 2018—Apr. 2020) including students and staff on a certain campus. There was no one outside this location. Participation was strictly voluntary, and participants did not receive any money. In Japan, annual health check-up for all employees and students are required by law, and the data, including serum alanine aminotransferase (ALT), aspartate aminotransferase (AST), lactate dehydrogenase (LDH), total bilirubin and serum creatinine, were used to rule out liver and kidney disease. Medical school staff and students are tested for HBV and HCV at the same time. All of these costs are paid by the university.

Subjects with known diabetes mellitus, hepatitis B or C virus infection, excess alcohol intake (> 20g/day), thyroid disease, and long-term drug therapy such as corticosteroids were excluded from this study. US and MRI examinations were performed on the same day.

This prospective study was approved by the research ethics committee of our institutional review board (Gunma University Graduate School of Medicine, Japan), and written informed consent was obtained from all participants. There were no relevant conflicts of interest.

### US-HRI

US examination was performed using a HI VISION Ascendus (Hitachi Ltd, Tokyo, Japan) unit equipped with a curved phased-array probe EUP-C715 (1–5 MHz). Imaging examinations and measurements were performed by a board-certificated diagnostic radiologist (ATT) with twenty years of experience. Instrument settings such as gain and depth were adjusted by the operator, depending on the body size of participants.

**Fig 1:**
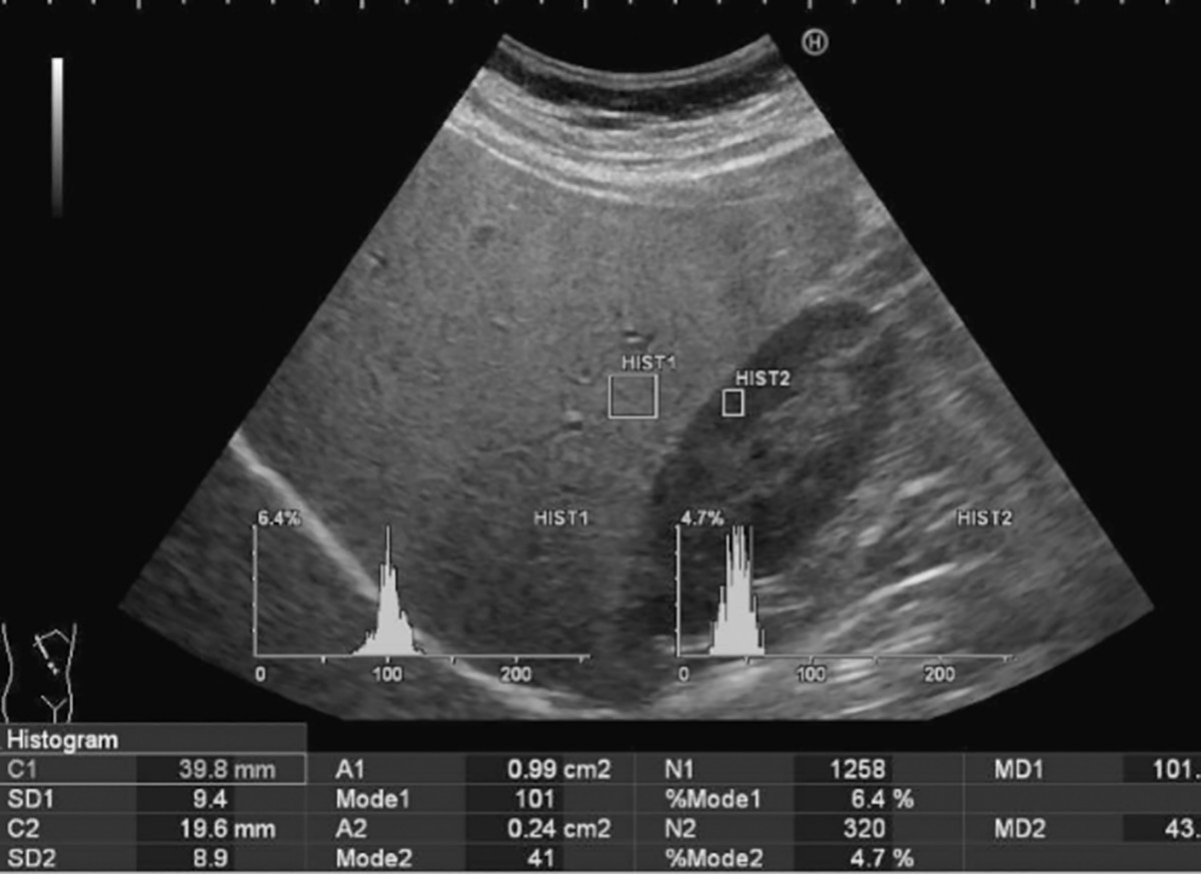
HRI measurement on a volunteer with mild hepatic steatosis (HRI = 2.33).

An image with the liver and right kidney in the same field of view was obtained in the left lateral decubitus position from the right sagittal or right intercostal approach. Regions of interest (ROIs) with a size of 100 mm^2^ in the liver parenchyma and 25 mm^2^ in the right renal cortex were selected (Fig 1). The ROIs were selected to avoid blood vessels and situated near the center of the image to be the same depth, gain, and mean gray-scale of the pixels [8]. If images of the liver and right kidney could not be obtained in the same field of view in the left lateral decubitus position, the liver and right kidney were imaged in the prone position from a right sagittal approach. US-HRI was calculated as the ratio of the echogenicity of the hepatic parenchyma to the echogenicity of the right renal cortex. This procedure was repeated five times with two ROIs on each scan. The mean of the three closest values was used with the difference between the values obtained being less than 0.2 [8].

### MRI-PDFF

**Fig 2:**
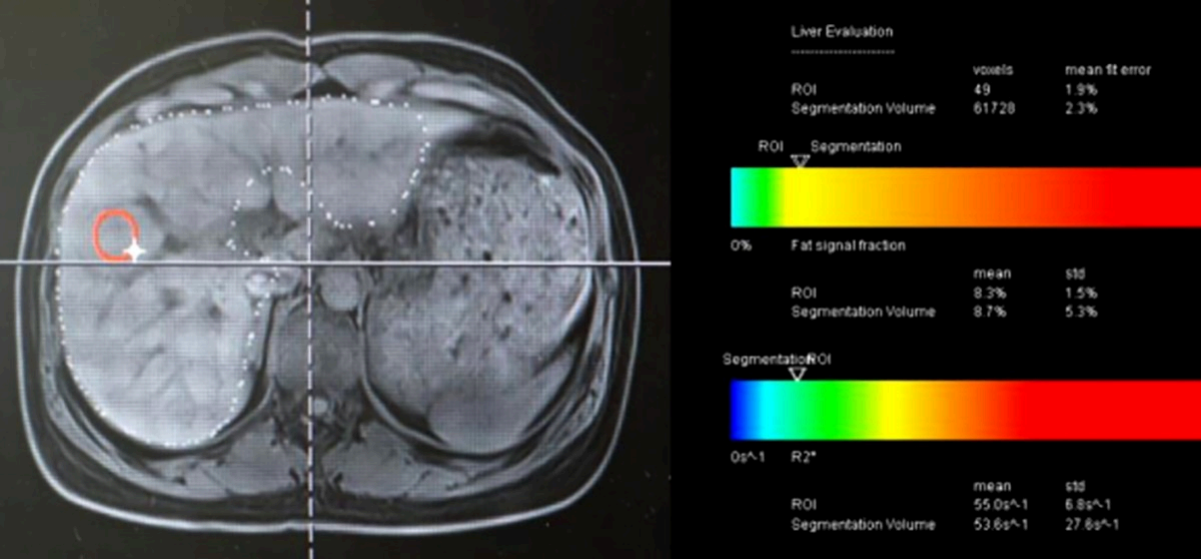
MRI-PDFF measurement on a volunteer with mild hepatic steatosis (MRI-PDFF = 8.3%).

The six-point Dixon technique was employed using modeling of a multi-echo adaptive fitting approach (LiverLab, Siemens Medical Systems, Erlangen, Germany) with a 3.0-Tesla magnet (MAGNETOM Skyra, Siemens Medical Systems, Erlangen, Germany). Multi-axial images were obtained by the three-dimensional gradient-recalled-echo (3D-GRE) pulse sequence with a 24-channel spine matrix coil and 18-channel body matrix coil. To estimate water and fat signals, six echoes with whole liver coverage were conducted in a single breath-hold (12 seconds). A short TR (9 ms) and a small flip angle (α = 40) were used in this pulse sequence with the aim to minimize T1 bias and T2*-effect. Other imaging parameters were: field of view (FOV) 350 mm, matrix 95 x 160, slice thickness 3.5 mm, echo time (TE) 1.12, 2.46, 3.69, 4.92, 6.15, 7.38 ms, parallel imaging factor of 2 x 2, and spatial resolution of 2 x 2 x 2 mm^3^. The Dixon sequence automatically generated series of water, fat, water percentage, fat percentage, goodness-of-fit, R2* map, T2* map, and fat fraction. ROIs were manually set in the right hepatic lobe to be as large as possible while avoiding margins, biliary tract, gallbladder, artifact, and large vessels. The goodness-of-fit was an indication of fitting residual errors of the fat percentage result, and MRI-PDFF were calculated as shown on Fig 2. The MR imaging and measurements were performed by a technologist (BVT) with 8 years of experience in MRI.

### MRS-PDFF

Immediately after MRI-PDFF measurements, a single-voxel MRS was performed to measure fat content as the reference standard. A high-speed T2-corrected multi-echo (HISTO) sequence was employed with a 15 seconds breath-hold. A stimulated echo acquisition mode (STEAM) was applied with the following parameters: voxel size of 30 mm x 30 mm x 30 mm (27 ml), TR of 3000 ms, 5 spectra at TE of 12, 24, 36, 48 and 72 ms, number of excitation (NEX) 1, and receiver bandwidth of 1200 Hz/Px. A voxel was placed in a homogeneous portion of the liver avoiding margins, biliary tract, gallbladder, artifact, and large vessels. On MRS, with the axial image active, the scroll nearest tool was used to select the coronal and sagittal image to the voxel position on the axial much the same as normal spectroscopy positioning.

**Fig 3:**
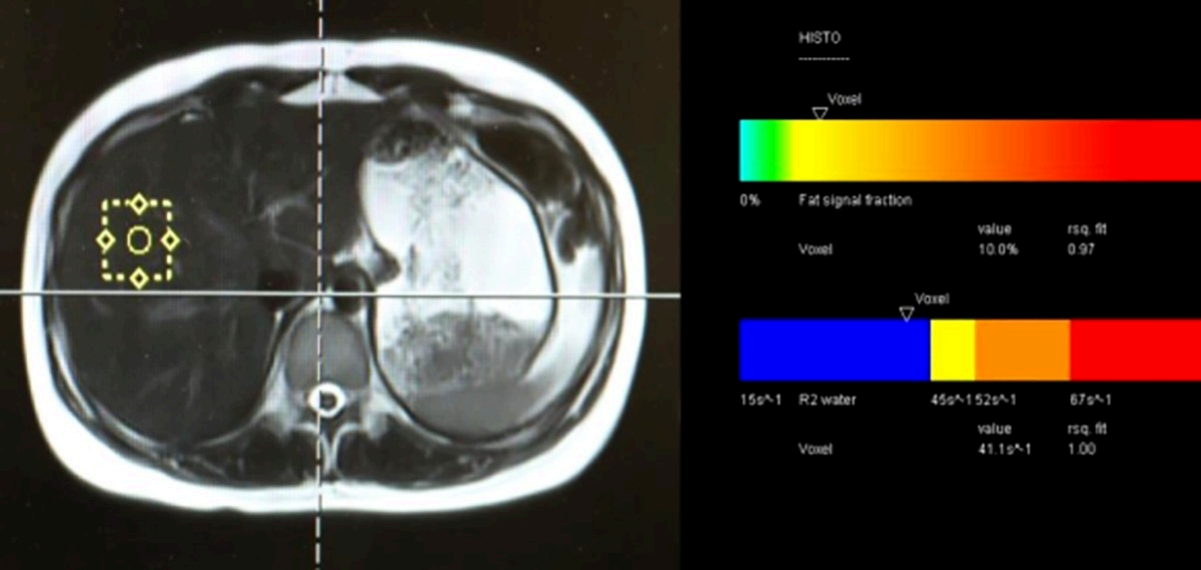
MRS-PDFF measurement on a volunteer with mild hepatic steatosis (MRS-PDFF = 10%).

Data were baseline corrected, phase-corrected, averaged and Fourier transformed. Levenberg-Marquardt curve fitting was performed using a combined Lorentzian-Gaussian model to calculate the area under the curve of fat and water peaks [11, 12]. MRS-PDFF was calculated as shown on Fig 3. The color bar map showed the amount of fat as a percentage. The measurement of MRS was performed by one technologist (KU) with 15 years of experience.

Measurements of US-HRI, MRI-PDFF and MRS-PDFF were separately performed without knowledge of the results of other measurements. However, as much as possible, the ROI for MRI-PDFF measurement was placed in the same position as the voxel in MRS-PDFF measurement in the right hepatic lobe, since the liver fat distribution may be inhomogeneous, potentially affecting the signal intensity. According to Szczepaniak and colleagues [13], grade 0 (normal), grade 1 (mild), grade 2 (moderate), and grade 3 (severe) were defined as corresponding to 0 - ≤5.56%, 5.56% - ≤10%, 10% - ≤20%, and >20% fat content, respectively. For MRS-PDFF, 5.56% fat was considered the cut-off value for this study.

### Statistical techniques

Pearson’s correlation was used to correlate the US-HRI and MRI-PDFF with MRS-PDFF. Receiver operating characteristic (ROC) curves including the area under curves (AUC) values were calculated to evaluate the accuracy of US-HRI and MRI-PDFF in determining hepatic steatosis. Optimal cut-off values giving sensitivity and specificity were computed by using Youden index.

The reproducibility of US-MRI and MRI-PDFF measurements was evaluated in 15 randomly selected subjects, who underwent two repeated measurements within an interval of 100 days to avoid the alteration of hepatic fat content over time [14]. Limits of agreement using the mean value of the two different measurements were calculated according to Bland-Altman analysis [15].

All analyses were conducted using the statistical software SPSS version 25.0 (SPSS Inc. Chicago, IL), and *p* < 0.05 were considered significant.

## Results

### Participants

A total of 52 participants (age, 31.5 ± 6.5 years [mean ± SD]; range, 20 to 50) matched the inclusion and exclusion criteria, with 30 men (25–50 years) and 22 women (20–35) included in this study. Body mass index (BMI) was 23.12 (± 3.62 kg/m^2^) according to the WHO formula.

### Diagnosis of hepatic steatosis based on MRS-PDFF

MRS-PDFF ranged from 1.0 to 16.7% (5.3±3.9% [mean±SD]). When the cut-off value was 5.56% on MRS-PDFF for the diagnosis of hepatic steatosis, sixteen subjects (30.8%) had mild to moderate hepatic steatosis. There were no subjects with severe hepatic steatosis.

### Correlations of US-HRI and MRI-PDFF with MRS-PDFF

**Fig 4:**
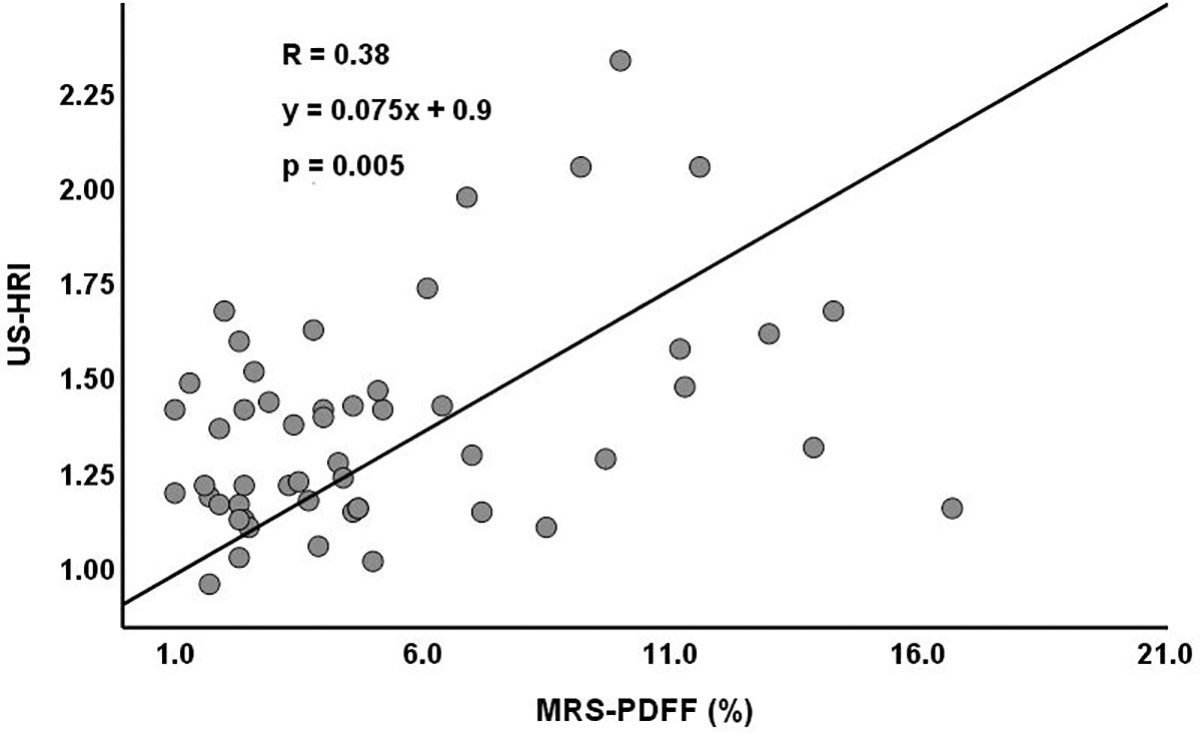
Correlation between US-HRI and MRS-PDFF.

**Fig 5:**
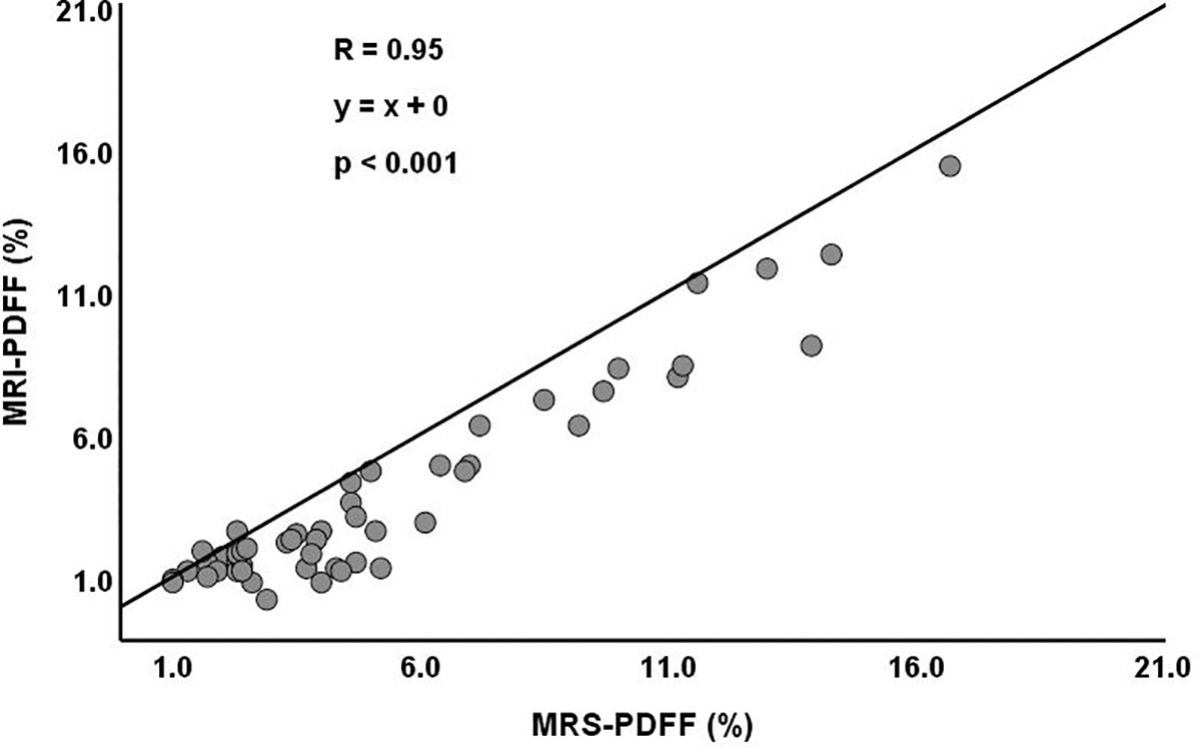
Correlation between MRI-PDFF and MRS-PDFF.

US-HRI ranged from 0.95 to 2.33 (1.4±0.3). The Pearson correlation coefficient between US-HRI and MRS-PDFF was significant but weak (*r* = 0.38, *p* = 0.005; Fig 4). MRI-PDFF ranged from 0.2 to 15.4% (3.8±3.5). The Pearson correlation coefficient between MRI-PDFF and MRS-PDFF showed excellent linear correlation (*r* = 0.95, *p*<0.001; Fig 5).

### Diagnostic accuracy

**Fig 6:**
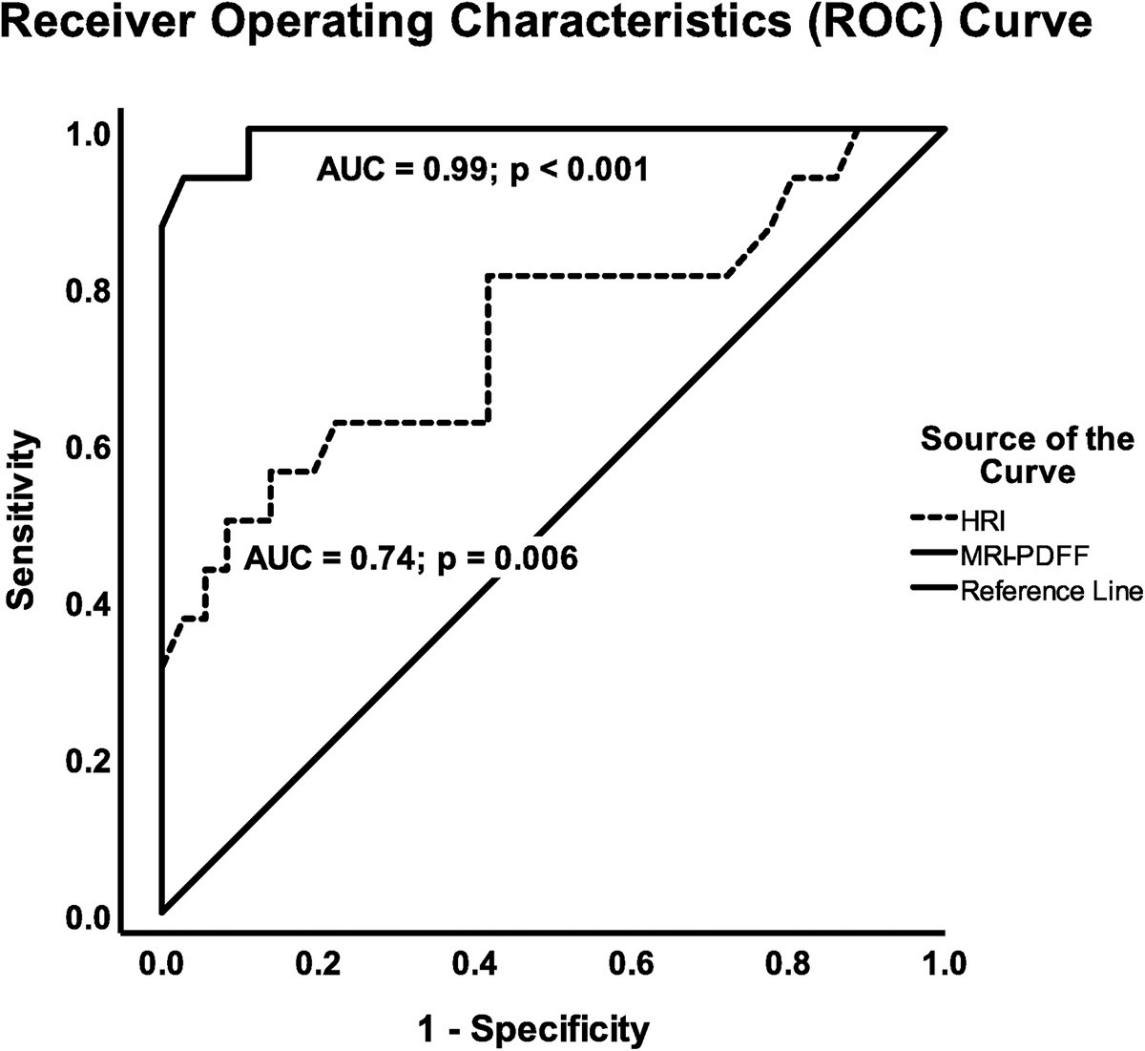
ROC curve using the reference of 5.56% as cut-off point in defining diagnostic performance.

With a 5.56% cut-off for MRS-PDDF, there were 16/52 participants who had mild to moderate steatosis. MRI-PDFF showed higher sensitivity (100%) and similar specificity (88.9%), compared to US-HRI (50% and 91.7%, respectively) for the diagnosis of hepatic steatosis. The cut-off values for MRI-PDFF and US-HRI were 2.75% and 1.54, respectively (Table 1). The AUC value of MRI-PDFF (0.99) was higher than US-HRI (0.74) (Fig 6). On ultrasound, the quantity of accuracy (ACC) was 78.85%. Meanwhile, the ACC of MRI-PDFF was 84.62%.

**Table 1:** Diagnostic performance of US-HRI and MRI-PDFF.

	MRI-PDFF	US-HRI
Sensitivity	1.0	0.5
Specificity	0.889	0.917
AUC	0.99 (*p*<0.001)	0.74 (*p* = 0.006)
Cut-off point	2.75 (%)	1.54
Quantity of accuracy	84.62%	78.85%

### Reproducibility

**Fig 7:**
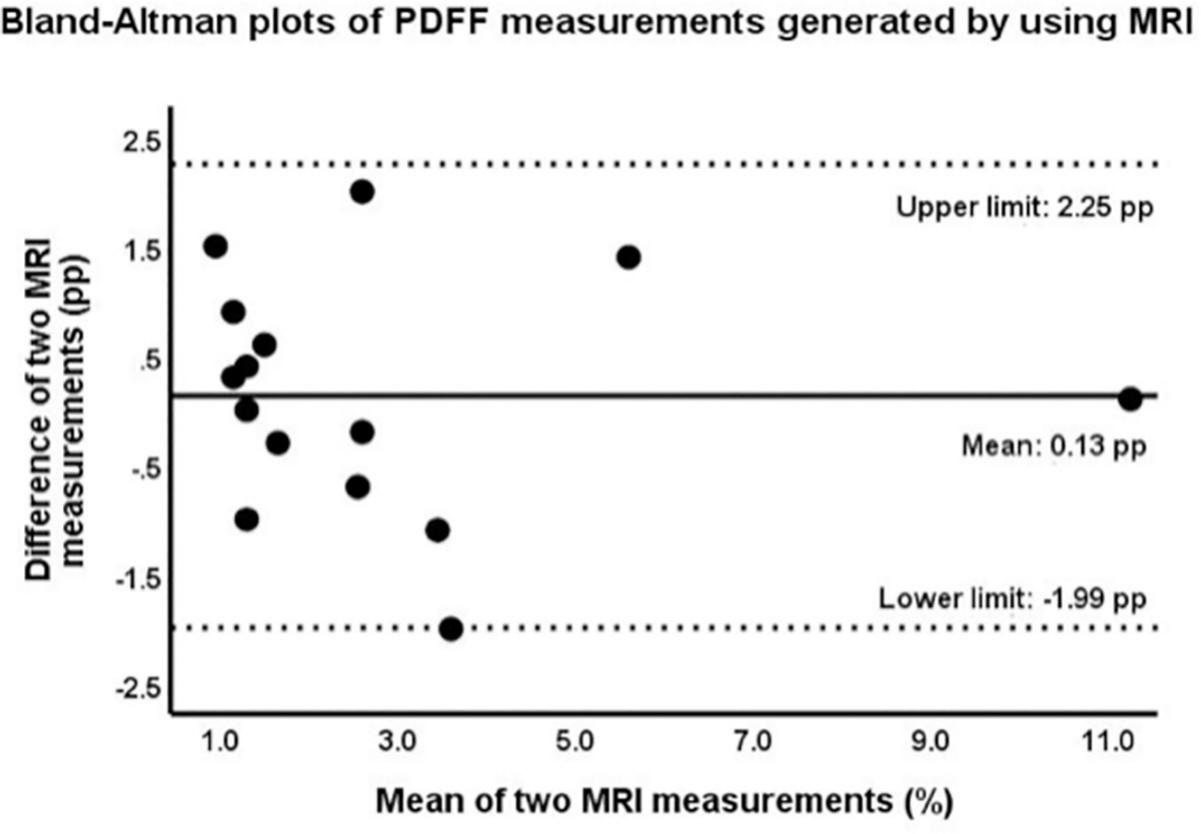
Bland-Altman plots for variability of PDFF measurements generated using MRI.

**Fig 8:**
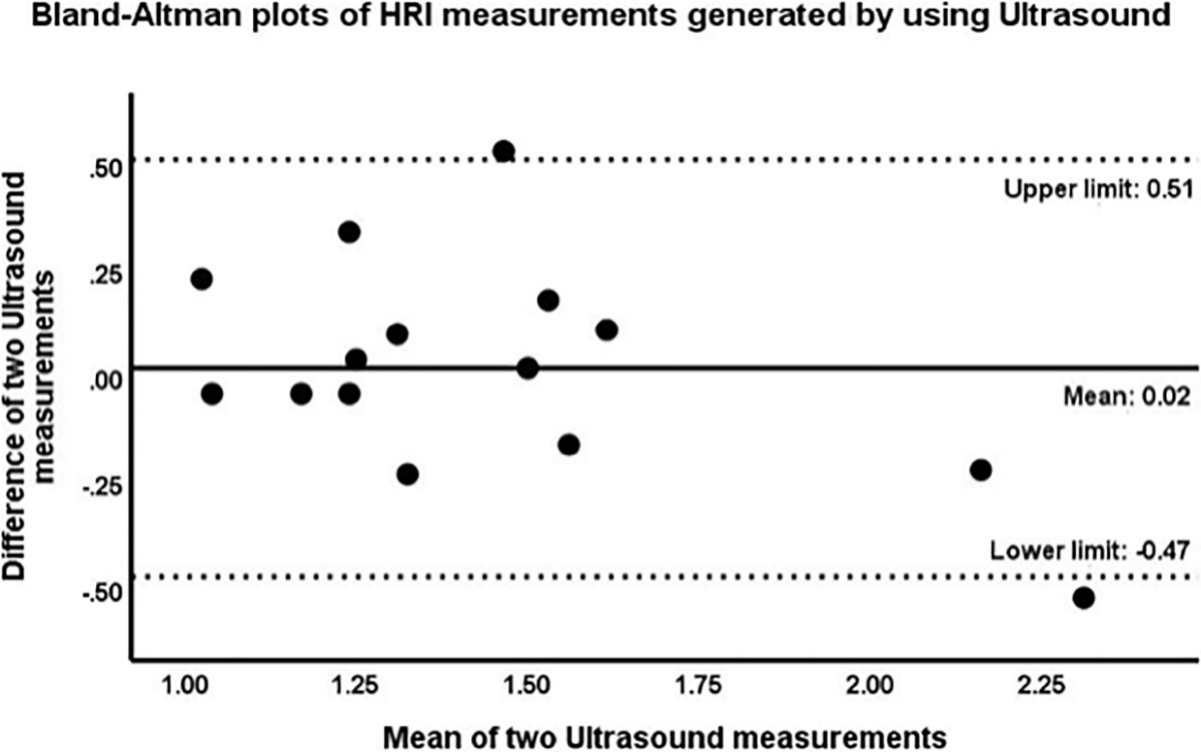
Bland–Altman plots for variability of HRI measurements generated using ultrasound.

The correlation coefficients for two repeated measurements of US-HRI and MRI-PDFF were 0.70 (p<0.001) and 0.85 (p<0.001), respectively. Bland-Altman analysis showed an excellent agreement between two measurements of MRI-PDFF with the mean of difference of 0.13 percentage points (pp) (limits of agreement [LOA], -1.99 pp and 2.25 pp). The mean of difference between two measurements of US-HRI was 0.02 (LOA, 0.47 and 0.51) (Figs 7 and 8).

The central line shows the mean of the differences between two PDFF measurements; the dashed lines show upper (mean + 1.96 SD) and lower (mean—1.96 SD) limits of agreement. Here, the mean difference is 0.13 pp, while the limits of agreement are -1.99 pp and 2.25 pp, indicating that 95% of the differences between these two measurements are within this range. The width interval is 4.24 pp.

The central line shows the mean of the differences between two HRI measurements; the dashed lines show upper (mean + 1.96 SD) and lower (mean—1.96 SD) limits of agreement. Here, the mean difference is 0.02, while the limits of agreement are -0.47 and 0.51, indicating that 95% of the differences between these two measurements are within this range. The width interval is 0.98.

## Discussion

In the current study, we found that MRI-PDFF showed excellent linear correlation with MRS-PDFF (the gold standard in this study), and its sensitivity for the diagnosis of hepatic steatosis was 100%, while that of US-HRI was 50%. The reproducibility of MRI-PDFF was also very good with a mean difference between two measurements of only 0.13 pp. MRI-PDFF is technically easier to implement than MRS-PDFF. The examination time is only 15 min., and does not require any special software package. To our knowledge, this was the first study directly comparing the reliability of MRI-PDFF and US-HRI in quantifying liver fat content.

According to a study comparing US-HRI and MRS-PDFF in 121 volunteers [8], there was a very good correlation between the two techniques (*r* = 0.89, *p*<0.001), thus it was concluded that US was valid enough for the identification, assessment and quantification of hepatic steatosis. On the other hand, in another study comparing US-HRI and MRI-PDFF in 34 overweight adolescents [16], there was only a moderate correlation between the two (*r* = 0.487, *p* = 0.003), and that report concluded that US can be used as a screening tool for non-alcoholic fatty liver diseases, but the diagnosis should be confirmed with MRI-PDFF. The disagreement between these findings is unexpected, since both MRS-PDFF and MRI-PDFF are measurement methods that use the difference in the resonance frequencies of water and lipid protons, and there should, theoretically, be no significant difference between the two measurements. However, while the two methods are based on the same physical principle (the small difference in resonance frequency between water molecule protons and fat molecule protons), actual signal processing is not the same. The Dixon method acquires signal when the water molecule protons and the fat molecule protos are in-phase and when they are in opposed-phase. Then, the sum or difference of these signals are calculated pixel-by-pixel. These signals are processed into images, and a ROI is selected for measurement. The in-phase and opposed-phase images are acquired by selecting different TEs on gradient echo (GRE) sequences [17]. In theory, for a given TE, all protons will be in-phase or in opposed-phase, but in reality, the local molecular environment of the protons is not completely homogenous, and this will, albeit slightly, alter the resonance frequency. In addition, technical limitations of hardware and static field inhomogeneity limit the accuracy of TE (ms) to about the second decimal point.

On the other hand, MRS measures water molecule protons and fat molecule protons directly and separately for a given voxel. No images, sums or differences are involved, and minor variations in frequency due to the state of protons become part of the distribution of frequency when graphed [18]. There is no spatial information, and a large voxel is needed for sufficient signal-to-noise ratio, but generally speaking, it is the most accurate method of measurement.

Additional factors, such as the variation of T1 relaxation time by TR, make it near impossible to make data acquisition completely identical while maintaining clinical feasibility. Given these factors, the different values are not surprising, and the difference in the slopes of the linear correlation graphs is also understandable.

These limitations notwithstanding, the results of the current study indicated that MRI-PDFF and MRS-PDFF were interchangeable, and MRI-PDFF, which is the simpler method, may be ideal in the clinical setting.

In the current study, the optimum cut-off value of MRI-PDFF for diagnosing fatty liver was 2.75%, with sensitivity and specificity of 100% and 88.9%, respectively. These results were consistent with a study of 94 subjects in determining the accuracy of MRS-PDFF using histopathologic analysis as the standard, showing sensitivity of 100% and specificity of 79% [19]. In a study investigating the accuracy of MRI in quantifying liver fat in 86 children, the authors found a slightly higher optimum MRI-PDFF threshold value of 5.1% with a sensitivity and specificity of 95% and 100%, respectively [20].

Since liver fat distribution may be inhomogeneous, the signal intensity on MRI may also be inhomogeneous. To the best of our konwledge, no imaging technique can adequately evaluate inhomogeneous fat distribution. When evaluating therapeutic efficacy, if fat distribution is not uniform, PDFF should be evaluated at exactly the same region before and after treatment. This is simple to accomplish on MRI because we can easily confirm the inhomogeneity of fat distribution visually. Although MRS-PDFF is an accurate measurement method, the inability to identify non-uniform fat distribution is a major drawback.

This study used US-HRI as a prevalent imaging technique for the diagnosis of hepatic steatosis in a routine setting. However, the current study showed only average agreement between two measurements, with an intra-observer correlation coefficient of 0.70 (*p*<0.001). Despite being a popular method, US-HRI has shown diverse results. According to data compiled and published by Chauhan and colleagues [21], threshold values varied from 1.24 to 2.02, sensitivity from 62.5% to 100%, and specificity from 54% to 96%. A common cause mentioned for the wide variation in ultrasound was its greater sensitivity to larger proportions of fat. Machine and operator dependence also commonly contributed to the wide variation of results [22].

The incomparability of US-HRI measurements from different machines or different operators limit the reliability of hepatic steatosis diagnosis from ultrasound measurements. Xia MF and colleagues proposed an improved method for comparing MRS with standardized US-HRI [23]. In this report, the authors tested the contribution of the standardization of the US-HRI using two types of US equipment, and reported high correlation coefficient between US-HRI and MR spectroscopy results. This technique is attractive and promising, but they only tested the standardization approach on two types of US equipment supplied by the same company (GE Healthcare). This makes it difficult to generalize this approach to US units from other suppliers, considering the differences in hardware and postprocessing procedures.

There are also several limitations for US-HRI and MRI-PDFF measurements in this study. First, the ROI was limited in size. In subjects with inhomogeneous liver fat distribution, even if multiple ROIs were averaged, there would be no guarantee that the fat content of the entire liver was measured accurately. ROIs were selected manually so the fat evaluation could never be entirely random or entirely objective. Second, the current research employed only one US machine. It is quite possible that results would vary among US units. Moreover, the discrepancy in post-processing algorithms in ultrasound and MRI scanners may limit the correlation between US and MRS. Third, using no histopathology for diagnosing hepatic steatosis as the reference standard may be a potential limitation due to the true prevalence of steatosis not being known with certainty among the participants of the present study. Finally, the measurement of the fat content in MR imaging was based on an available software, and the parameters were not changed from the manufacturer’s settings. There was no comparison of parameters to optimize assessment. Additionally, the noise performance was also not examined, leading to the SNR-effect being ignored on the image reconstruction.

## Conclusions

In conclusion, with MRS-PDFF ≥5.56% defined as the gold standard of fatty liver disease, AUCs, cut-off values, sensitivities and specificities of US-HRI and MRI-PDFF were 0.74, 2.75%, 50%, 91.7% and 0.99, 1.54, 100%, 88.9%, respectively. The intraclass correlation coefficients (ICCs) of MRI-PDFF were excellent (0.85), compared to US-HRI (0.70). Therefore, MRI-PDFF was a more reliable technique to for the diagnosis of hepatic steatosis.
